# Primer on the Immune System

**DOI:** 10.35946/arcr.v37.2.02

**Published:** 2015

**Authors:** Martin J. Spiering

**Affiliations:** Martin J. Spiering, Ph.D., ELS, is a senior staff member with CSR, Incorporated, and contributing editor to *Alcohol Research: Current Reviews*.

The human body regularly encounters and combats many pathogenic organisms and toxic molecules. Its ensuing responses to these disease-causing agents involve two interrelated systems: innate immunity and adaptive (or acquired) immunity. Innate immunity is active at several levels, both at potential points of entry and inside the body (see figure). For example, the skin represents a physical barrier preventing pathogens from invading internal tissues. Digestive enzymes destroy microbes that enter the stomach with food. Macrophages and lymphocytes, equipped with molecular detectors, such as Toll-like receptors (TLRs), which latch onto foreign structures and activate cellular defenses, patrol the inside of the body. These immune cells sense and devour microbes, damaged cells, and other foreign materials in the body. Certain proteins in the blood (such as proteins of the complement system and those released by natural killer cells, along with antimicrobial host-defense peptides) attach to foreign organisms and toxins to initiate their destruction.

When a pathogenic organism or toxin does gain a foothold in the body, the defenses furnished by the innate immune system are reinforced by those of the adaptive immune system. Compared with innate immunity, adaptive immunity is a more evolved and complex system consisting of both cells and proteins. These adaptive immunity agents specifically target and destroy the invading pathogen. Within days or weeks, the adaptive immune system manufactures antibodies tailored to the pathogenic invader to halt its spread. This process, known as the humoral response or antibody-mediated immune response, relies on specific cell types, called B cells, which produce antibodies. In parallel, this response activates lymphocytes, including T cells, programmed with information to detect surface molecules specific to the invader—a second type of adaptive immunity called cellular immunity. A hallmark of adaptive immunity is that it can store—via production of specialized T and B cells—a memory of the pathogen’s unique molecular structures allowing for a more rapid response to future invasions by the same pathogen.

The expanded glossary below presents the main features of and mechanisms and players in the innate and adaptive immune systems that are relevant to this special issue of *Alcohol Research: Current Reviews*.

## The Innate Immune System

Responses of the innate immune system to acute or persistent infection or injury typically manifest as inflammation. The primary purpose of the inflammation is to contain the infection, enable rapid access of immune cells and proteins to the infection site, and promote healing once the pathogen(s) has been cleared. This process involves multiple cytokines and types of immune cells. Many of the cells of the innate immune system are phagocytes: cells that ingest other cells or cellular debris through a process called phagocytosis, which neutralizes harmful agents. In phagocytosis, the immune cells engulf microorganisms or foreign particles and inactivate them in an intense chemical shower of reactive oxygen species called the respiratory burst.

Innate immune cells have various functions, including the following.

**Granulocytes** are white blood cells (i.e., leukocytes) characterized by the presence of granules in their cytoplasm. Granulocytes include the following cell types:

*Neutrophils* are the most abundant granulocytes and also the most abundant type of white blood cell, reaching concentrations of up to 5 million cells per milliliter in the blood. Neutrophils normally circulate in the blood and, upon injury or infection, quickly move to the affected site. They thereby follow chemical signals consisting of *cytokines* and *chemokines* to the site where they are among the first immune cells to arrive. Neutrophils detect pathogens via TLRs and directly attack them, for example, through phagocytosis. Neutrophils also release extracellular traps composed of DNA and antimicrobial peptides that ensnare and kill microbes. Thus, neutrophils represent an important first-line defense against invading microbes.

*Basophils* originate from bone marrow and circulate in the blood; they are the least abundant white blood cells. Upon activation by proteins, they move to an injured or infected site. Similar to *mast cells*, basophils also sometimes cause inflammatory responses such as allergic reactions. Basophils release the anticoagulant heparin and the vasoactive compounds histamine and serotonin, which reduce blood clotting and contribute to wound swelling typical of inflammations, respectively.

*Eosinophils* develop and mature in bone marrow and then also circulate in the blood. They are activated, for example, by lymphocytes of the adaptive immune systems, and they are crucial for combating larger parasites that cannot be phagocytosed, such as protozoans. Eosinophils also help fight other types of infections.

*Mast cells* reside in connective tissues and mucous membranes and aid in wound healing and also in defending against pathogens. When activated by pathogens or allergens such as pollen, mast cells rapidly release protein-carrying granules rich in both histamine and heparin, molecules involved in inflammation. Mast cell activation often underlies adverse immune responses such as allergies, arthritis, and anaphylactic shock.

**Monocytes** are the largest cells of the innate immune system. They mature in bone marrow and then circulate through the blood. Half of them are stored in the spleen and the other half in other locations throughout the body. Monocytes are precursors for two other innate immune system cells: *macrophages* and *dendritic cells*.

*Macrophages* are cells that search for and phagocytose pathogens. Upon exiting blood and entering tissues, *monocytes* develop into macrophages. They help remove excess, damaged, or dead cells marked by surface proteins for elimination. “Resident” macrophages inhabit specific locations or organs that are prone to infections, such as the lungs and liver, or serve in hubs, such as the spleen, for rapid deployment to injured or infected sites. Examples include Kupffer cells, macrophages residing in the liver, and microglia, residing in the central nervous system. Macrophages carry on their surface several TLRs that are activated by pathogen- or damage-associated molecular patterns—this activation stimulates the macrophages to phagocytose pathogens or damaged cells or to secrete *cytokines* to activate and recruit additional immune cells. Macrophages contribute to wound healing, help control immune responses and other cells of the innate immune system, and also stimulate adaptive immunity (see below).

*Dendritic cells* act as messengers between the innate and adaptive immune systems. They reside in tissues exposed to the external environment, including the skin and the linings of the nose, lungs, stomach, and colon. Like *neutrophils* and *macrophages*, they detect foreign invaders via TLRs. Upon encountering a pathogen, dendritic cells ingest (i.e., endocytose) it or its products and attach pieces of the pathogen (i.e., antigens) to their cell surface on a protein assembly called the major histocompatibility complex II (MHC II). The dendritic cells then migrate to the lymph nodes where they activate *T cells* and *B cells* by presenting the pathogen’s antigens to them. Dendritic cells are the most potent of several types of antigen-presenting cells, which effectively jumpstart the adaptive immune response.

**Natural killer cells (NK cells)** rapidly respond to the presence of virus-infected and tumor cells and destroy them with proteolytic enzymes and cytotoxic proteins that destabilize the cells’ membranes and induce apoptosis. NK cells recognize stressed cells in the absence of the chemical triggers other immune cells need to mount an immune response. Although traditionally classified along with innate immune cells, some evidence of immunological memory in NK cells (see table) suggests that these cells are also affiliated with adaptive immunity.

**The complement system** consists of more than 30 blood-borne proteins produced in the liver. These proteins help or “complement” the killing of pathogens by antibodies. The complement system triggers a biochemical cascade in which foreign cells are first opsonized (i.e., coated) with complement proteins, weakening or rupturing (i.e., lysing) their cell walls. The action of complement also attracts other immune cells such as *macrophages* and *neutrophils*, along with antibodies, to the site of infection.

## The Adaptive Immune System

The cells and structures of all organisms display unique antigens, which are molecules characteristic only to them. During the development of the immune system, adaptive immune cells originating from lymphocytes differentiate to recognize specific antigens, and the entire complement of this antigen specificity enables recognition of all possible antigens. As rearrangements within the genes in the immune cells occur during this developmental process, antigens present in the host (self-antigens) interact with the emerging cell population to eliminate those adaptive immune cells that would attack the host, while retaining only those cells that will target any non–self-antigens.

The functions of cells of the adaptive immune system are as follows.

**B lymphocyte cells** display an enormous variation in the cells they target—the blood and lymphatic systems contain millions of B cells, produced early in the body’s development, which differ in the type of antibody they produce in response to the antigens they recognize. Each B cell carries a cell-surface receptor designed to fit a specific antigen on the pathogen. B cells scan for pathogens (such as viruses and bacterial toxins), and on encountering a pathogen whose antigen fits its receptor, a B cell will start to make copies of itself (i.e., proliferate). The proliferating B cells grow into a colony of plasma cells producing and secreting antibodies that block the pathogen from gaining access to healthy cells. After the infection has resolved, some of these plasma cells may persist for 50 years or longer as memory B cells, which contribute to immunological memory and can respond quickly by producing antibodies if they encounter the same pathogen again.

**T lymphocyte cells** mainly target cells of the body that have been invaded by pathogens such as viruses, or that show abnormal molecular patterns on their surface associated with cancerous growth or necrosis. T cells do not produce antibodies and they mature in the thymus. They may be broadly divided into three groups: helper T cells, cytotoxic T cells, and regulatory T cells.

*Helper T cells* (also known as CD4^+^ cells) represent a key cell type in adaptive immunity and consist of four groups. Th1 and Th2 cells are involved in defenses against intra- and extracellular pathogens and in autoimmune and allergic responses, respectively; a recently identified helper T cell, Th17, represents another CD4^+^ cell group involved in neutralizing extracellular microbes and also has been shown to be involved in chronic inflammation and autoimmune disease. Regulatory T (Treg) cells represent a fourth group that helps to check responses of effector T cells and suppress pro-inflammatory pathways—for example, when an infection has resolved. These cells also keep the immune system in check when there is no infection, preventing immune cells from attacking the normal cells of the body.

The T helper cells do not attack pathogens directly, but activate other immune system cells, including *B cells*, *killer T cells*, and *macrophages*. They are activated by antigens presented on, for example, *dendritic cells* and *B cells*. Each helper T cell, derived from cells produced early in the body’s development by the above-mentioned differentiation mechanism, has T-cell receptors on its surface that recognize a specific antigen attached to MHC II of the presenting cells. On encountering an immune cell that presents an MHC II–bound antigen that matches the helper T cell’s receptor, the helper T cell is activated and begins to proliferate. Some of these proliferating cells become memory helper T cells that contribute to immunological memory and respond quickly to future infections by the same pathogen. The others become effector helper T cells, which release *cytokines* to attract other immune cells, such as *macrophages*, *B cells*, and *cytotoxic T cells*, or regulate the activity of these cells.

*Cytotoxic or killer T cells* (also known as CD8^+^ cells) search for and destroy cells infected with viruses or other pathogens or for cells that are damaged or abnormal such as cancer cells. Like *helper T cells*, cytotoxic T cells have T-cell receptors. These receptors bind to MHC I, a protein complex that is present on the surface of all cells in the body. When a microbe or virus infects a cell or a cell becomes cancerous, fragments of damaged proteins are transported to the cell surface and are presented on MHC I. A cytotoxic T cell whose receptor fits an antigen presented on MHC I binds to the antigen, resulting in activation of the T cell. Activated cytotoxic T cells begin to proliferate into memory cytotoxic T cells or effector cytotoxic T cells. The latter cells bind to MHC I on the antigen-presenting cells and destroy them, whereas the former contribute to immunological memory of the activation event.

## Signaling in Innate and Adaptive Immunity

**Cytokines** are small proteins that help immune cells to communicate; they are secreted from immune cells on contact with a pathogen- or damage-associated molecular pattern or with an antigen. Many cells of the innate and adaptive immune systems release cytokines, which activate or suppress the activity of other immune cells by binding to specific receptors on these cells. Cytokines help regulate virtually all immune processes, affect the balance between humoral and cellular immunity, and help control the growth and maturation of many immune cells. They include *chemokines*, *interferons*, *interleukins*, and *tumor necrosis factor*.

*Chemokines* represent cytokines whose action on the receptors of immune cells (i.e., leukocytes) promotes movement (i.e., chemotaxis) toward the source of the chemokines; chemokines thus attract the immune cells to, for example, sites of inflammation or injury.

*Interferons* are cytokines released by cells (especially leukocytes) interacting with viruses, other pathogens, or toxic proteins; they bind to and activate specific receptors on neighboring cells. This activation leads to increased transcription of genes for proteins that increase the cells’ resistance to viral infection. Interferons also inhibit activation of *B cells* and increase the cytotoxicity of *NK cells*. Interferons are represented by three distinct classes (α, β, and γ), each of which is characterized by specific functions and is produced by specific cells (e.g., by leukocytes, fibroblasts, and *lymphocytes*).

*Interleukins* (*ILs*) are produced by leukocytes, *lymphocytes*, and even non-immune cells (in some circumstances). ILs include both cytokines and *chemokines*. Low concentrations of these proteins mainly facilitate localized communication among leukocytes in inflammation, such as promoting the production of chemokines to recruit additional immune cells. At higher concentrations, some ILs (e.g., IL-1) enter the blood stream and act as endocrine hormones, producing fever and stimulating production of immune proteins in the liver.

*Tumor necrosis factor* α (*TNF*α) is a major pro-inflammatory cytokine. It primarily is produced by macrophages and promotes inflammation both during infection and in dysregulated immune responses, such as those active in degenerative diseases (e.g., arthritis). By binding to and activating its specific cell receptor, TNFR (i.e., cluster of differentiation 120 [CD120]), TNFα activates several transcription factors such as nuclear factor κB, which upregulates expression of pro-inflammatory genes. TNFα also induces cell death (i.e., apoptosis) and necrosis in some cell types.

## Conclusion

The innate and adaptive immune systems have distinct roles in combating infections and pathogenic cells, and both systems have some modest functional overlap. Whereas innate immunity represents a relatively non-specific and first-line defense against microbes and parasites, adaptive immunity encompasses a highly evolved assemblage of sophisticated defense mechanisms that specifically target groups of related or individual pathogens. The innate immune system blocks entry of pathogens by physical (e.g., skin) and physiological (e.g., pH, nucleases, proteases, and host-defense peptides) means. If a pathogen succeeds in breaching these initial barriers, detection of the pathogen by innate immune cells stimulates inflammation that attempts to isolate infected cells and tissues and to inactivate the invading pathogen. If this initial inflammatory response does not eliminate the pathogen, the adaptive immune system comes into play.

The cells of the adaptive immune system translocate to the site of infection and begin to inactivate, for example, free virus particles (by way of *B cells*) and to destroy virus-infected or damaged cells (by way of *T cells*), or help eliminate other pathogens such as bacteria, fungi, or larger parasites. Formation of B and T memory cells then guards against future attack by the same pathogen.

Current research still is untangling the complex interactions between these two immune systems and studying the functions of the many proteins and chemical signals involved.

## Figures and Tables

**Figure:**
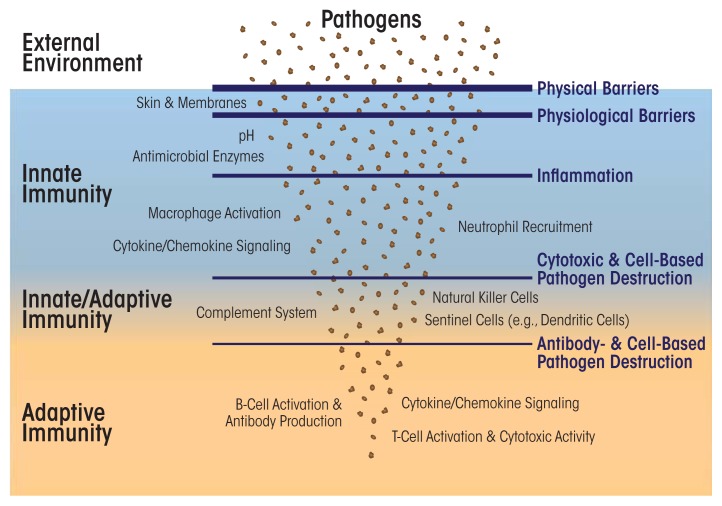
Overview of the immune system. Innate immunity encompasses several non-specific protective mechanisms against infection, including physical and physiological barriers, cells (e.g., macrophages and neutrophils) that detect and attack other cells carrying pathogen-associated molecular patterns, and small proteins that signal pathogen invasion (i.e., cytokines and chemokines) or short peptides that directly attach to and restrict microbial pathogens. The adaptive immune system comprises specialized cells (e.g., B and T cells) and proteins (i.e., antibodies) that detect and eliminate specific pathogens and also uses cytokine/chemokine signaling to recruit additional immune cells. Several cells in adaptive immunity (i.e., memory B and T cells) can store immune memory of a pathogenic invasion. The complement system, along with natural killer cells and dendritic cells, straddles both innate and adaptive immunity.

**Table:** Components of the Immune System

Innate Immunity	Adaptive Immunity
Immune responses are largely non-specific, e.g., via Toll-like receptors (TLRs)	Immune responses specifically target pathogens via its antigens detected by specific immune cell receptors
Comprises a variety of defense mechanisms, i.e., physical/physiological barriers, lytic enzymes, reactive oxygen species, isolation of diseased tissues, and cell- and protein-mediated immunity	Involves mainly cell- and protein-mediated immunity
Immediate response to pathogenic challenge	Lag time between pathogen detection and response
No immunological memory (with some evidence for immune memory in NK cells)	Activation leads to immunological memory
Often underlying chronic inflammation in allergies and degenerative diseases (e.g., Alzheimer’s disease, rheumatoid arthritis)	Often underlying autoimmune diseases in which self/nonself recognition is impaired, causing adaptive immune cells to attack the body’s own cells (e.g., in type I diabetes, autoimmune hepatitis)
Present in all eukaryotes (including plants, which, however, use different mechanisms and molecules in innate immunity)	Present in jawed vertebrates with emerging evidence of related immune mechanisms in jawless vertebrates and some invertebrates
